# Hsa_circ_0043265 Restrains Cell Proliferation, Migration and Invasion of Tongue Squamous Cell Carcinoma via Targeting the miR-1243/SALL1 Axis

**DOI:** 10.3389/pore.2021.587130

**Published:** 2021-02-03

**Authors:** Cuijuan Qian, Yisheng Yang, Tianchen Lan, Yichao Wang, Jun Yao

**Affiliations:** ^1^ Institute of Tumor, School of Medicine, Taizhou University, Taizhou, China; ^2^ Department of Medical Laboratory, Taizhou Central Hospital, Taizhou University Hospital, Taizhou, China

**Keywords:** circ_0043265, miR-1243, SALL1, circRNAs, tongue squamous cell carcinoma

## Abstract

Increasing evidence has displayed critical roles of circular RNAs (circRNAs) in tongue squamous cell carcinoma (TSCC). Hsa_circ_0043265 (circ_0043265) has been identified as a tumor suppressor in various tumors. Nevertheless, the critical roles of circ_0043265 in the initiation and progression of TSCC are yet to be fully elucidated. In our study, RNA and protein expressions were detected via qRT-PCR and Western blot. Cell proliferation, migration and invasion were evaluated via CCK-8 and transwell assays. The interactions between circ_0043265, miR-1243 and SALL1 were analyzed via bioinformatics analyses, RNA pull-down and luciferase assays, respectively. The current study demonstrated that circ_0043265 expression was downmodulated in TSCC tissues and cell lines (SCC25, SCC15, SCC9 and Cal27). Functionally, circ_0043265 overexpression led to an attenuation of cell proliferation, migration and invasion of SCC25 and Cal27 cells. Mechanistically, circ_0043265 acted as a competing endogenous RNA (ceRNA) via competitively sponging miR-1243, and restoration of miR-1243 rescued the inhibitory effects of circ_0043265 on cell proliferation, migration and invasion of SCC25 and Cal27 cells. Finally, it was observed that spalt like transcription factor 1 (SALL1), a potential target of miR-1243, was positively modulated via circ_0043265 in SCC25 and Cal27 cells, and SALL1 knockdown reversed the inhibitory effects of circ_0043265 on SCC25 and Cal27 cells. Collectively, the current study demonstrated that circ_0043265 was downmodulated in TSCC and was identified as a ceRNA that restrained the cell proliferation, migration and invasion of SCC25 and Cal27 cells via modulating the miR-1243/SALL1 axis.

## Introduction

Tongue squamous cell carcinoma (TSCC) is one of the most common malignant tumors in head and neck region, and is characterized via a high rate of regional lymph node metastasis and recurrence [1]. In most case, TSCC is clinically silent at the early stage, with symptoms only appearing at the late stage [1,2]. Although some progress has been achieved in the development of surgical and combination therapies, therapeutic options for TSCC still remain very limited [2]. Furthermore, the complexity of carcinogenesis and the lack of effective clinical biomarkers are considered as the main obstacles to improve TSCC patients’ prognosis [3]. Therefore, elucidation of the complicated molecular signatures and mechanisms of TSCC may aid in the diagnosis, treatment and prognosis of TSCC.

Circular RNAs (circRNAs) are a class of noncoding RNAs (ncRNAs) that form a closed circular structure through covalent bonds [4,5]. Compared to linear RNAs, circRNAs are more conservative and stable [4]. Recently, increasing evidence has demonstrated that aberrant expression of circRNAs is implicated in the initiation and progression of various tumors, where they can act as oncogenes or tumor suppressors [5,6]. Furthermore, circRNAs are involved in the modulation of the expression and function of downstream target genes, either post-transcriptionally or via chromatin modulation in TSCC initiation and progression [7–11]. Nevertheless, only a small number of circRNAs have been functionally characterized in TSCC at present, and the mechanisms underlying their biological functions are yet to be fully elucidated. Hsa_circ_0043265 (circ_0043265) is a newly discovered circRNA, and a recent study has identified circ_0043265 as a tumor suppressor in non-small cell lung cancer (NSCLC) [12]. Nevertheless, the expression profile, clinical significance, biological function and mechanism of circ_0043265 in TSCC have not been fully investigated.

The aims of the current study were to identify the expression, function and mechanism of circ_0043265 in TSCC. It was demonstrated that circ_0043265 was downmodulated in TSCC tissues and cell lines, circ_0043265 overexpression restrained the cell proliferation, invasion and migration of TSCC cells, and circ_0043265 acted as a ceRNA to positively modulate the expression of SALL1 via sponging miR-1243 in TSCC cells. Collectively, these results demonstrated the tumor suppressor role of circ_0043265 in the initiation and progression of TSCC.

## Material and Methods

### Clinical Specimens

The current study was approved by the Ethical Committee of Taizhou University Hospital (2019.02.26). The pathologically diagnosed TSCC patients enrolled in the study provided their informed consent. A total of 40 pairs of TSCC and their adjacent non-tumor tissues were collected from the Taizhou University Hospital.

### Cell Culture

Human TSCC cell lines (SCC25, SCC15, SCC9 and Cal27) were obtained from American Type Culture Collection (ATCC, Manassas, VA, USA), and human oral keratinocytes (HOK) were prepared from outgrowth cultures of mucosal biopsies with appropriate ethical approval. All cells were cultured in Gibco^®^ Dulbecco's Modified Eagle's medium (DMEM; Gibco; Thermo Fisher Scientific, Waltham, MA, USA) supplemented with 10% fetal bovine serum and 100 U/ml penicillin and streptomycin at 37 °C in a humidified atmosphere of 5% CO_2_.

### QRT-PCR (Quantitative Real-Time RT-PCR)

RNA/miRNA extraction and qRT-PCR were performed as described in the previous studies [12,13]. In brief, total RNA was extracted form TSCC cells, HOK or tissues of TSCC patients, respectively. RNA samples were then reverse transcribed into cDNA with PrimeScript™ RT Reagent kit (TaKaRa, Kyoto, Japan). Then, SYBR Green PCR Master Mix (TaKaRa, Kyoto, Japan) was used to detect and quantify circ_0043265, miR-1243, SALL1, GAPDH and U6 mRNA expression. The relative expression levels were calculated using the 2^−ΔΔCt^ method. Their primers were purchased from Sangon (Shanghai, China) and sequences as described previously [12,13] are listed in Table 1.

**TABLE 1:** QRT-PCR primer sequences.

Name	Primer sequence
circ_0043265	F: 5′-CAACGCAGGCATCAGAAGATT-3′ R: 5′-AGGAAGGCCACTTCATAAGTCTG-3′
miR-1243	F: 5′-TAGGAGTGAAATAAAGGTCCATCTC-3′ R: 5′-CCAAAGCAAAGTAATAAATAGGCAG-3′
SALL1	F: 5′-TGATGTAGCCAGCATGT-3′ R: 5′-AAAGAATTCAGCGCAGCAC-3′
GAPDH	F: 5′-GCACCGTCAAGGCTGAGAAC-3′ R: 5′-GCCTTCTCCATGGTGGTGAA-3′
U6	F: 5′-GCTTCGGCAGCACATATACTAAAAT-3′ R: 5′-CGCTTCACGAATTTGCGTGTCAT-3′

### Plasmid Construction and Transfection

Lentivirus circ_0043265 overexpression vector (Lv-circ_0043265) and its negative control (Lv-NC, lentivirus-negative control), miR-1243 mimic and its negative control (control mimic), siRNA against SALL1 (SALL1-siRNA) and its negative control (si-NC) were all provided by GeneChem (Shanghai, China). The sequences of siRNA, miRNA mimic and their controls were listed as follows: SALL1-siRNA: sense, 5′-CCA GAU CUA UGA ACU ACA ACA-3′; antisense, 3′-UUG UAG UUC AUA GAU CUG GGG-5′. si-NC: sense, 5′-UUC UCC GAA CGU GUC AGG UTT-3′; antisense, 3′-ACC UGA CAC GUU CGG AGA ATT-5′. miR-1243 mimic: 5′-AAC UGG AUC AAU UAU AGG AGU G-3′, and its control mimic: 5′-GUG GAU AUU GUU GCC AUC A-3′. Transfection was performed as previously described [12–14]. SCC25 and Cal27 cells were transfected with 100 ng of the vector or 50 nM of the indicated oligonucleotide by Lipofectamine™ 2000 (Invitrogen, CA, United States).

### Cell Proliferation Assay

The CCK-8 (Cell Counting Kit-8) kit (Beyotime, Shanghai, China) was used in this assay. 5,000 cells were seeded into each well of 96-well plates. After cell transfection, 10 μL of the CCK-8 solution was added to each well of cells. After 1 h of incubation, the absorbance of each well was measured using a microplate reader (Bio-Rad, Hercules, CA, United States).

### Transwell Assay

SCC25 and Cal27 cells were suspended in serum-free DMEM at a density of 4×10^5^ cells/ml. Then, cells (250 µL) were pipetted into the upper chamber coated with (for the invasion assay) or without (for the migration assay) Matrigel (BD Biosciences, San Diego, CA, United States). DMEM with 15% FBS (600 µL) was added to the lower chamber. For cell migration assay, cells were allowed to migrate for 24 h, and then cells in the upper chamber which were non-migrated were removed with a cotton swab, while cells on the bottom surface of the upper chamber were fixed in 100% methanol and stained with 2.5 µM 4′,6-diamidino-2-phenylindole (DAPI; Abcam, Cambridge, MA, United States) for 30 min. Finally stained cells were counted using a fluorescence microscope (Eclipse 80i; Nikon, Tokyo, Japan) in five random fields. For cell invasion assay, cells were allowed to invade for 48 h, and invaded cells were fixed in 70% ethanol and then stained with 0.1% crystal violet for 30 min. The stained cells were lyzed with 200 μL of lysis reagent. Finally, 100 μL of lysate was pipetted into a 96-well plate to get OD560 nm absorbance via a microplate reader (550; Bio-Rad, Hercules, CA, United States).

### Western Blotting

After cell transfection, proteins were extracted using RIPA buffer (Beyotime, Shanghai, China) and quantified using a bicinchoninic acid (BCA) protein assay kit (Beyotime, Shanghai, China). Then, 30 µg/lane protein was separated via SDS-PAGE and transferred to PVDF membranes. The PVDF membranes were blocked at room temperature and then incubated overnight at 4 °C with rabbit-anti-human SALL1 (1:1,000; cat.no.ab41974; Abcam, Cambridge, MA, United States) and GAPDH (1:1,000; cat.no.ab8245; Abcam, Cambridge, MA, United States) primary antibodies. The next day, membranes were incubated with a specific secondary antibody conjugated to horseradish peroxidase for 2 h at room temperature, and then the signals were detected using an enhanced chemiluminescence system kit (Pierce, Waltham, MA, United States) and visualized using an LAS-4000 imaging system (Fujifilm Holdings Corporation, Tokyo, Japan).

### Luciferase Assay

Luciferase assay was performed as described previously [14]. Briefly, the interactions between circ_0043265 and miR-1243 or SALL1 were predicted by Circular RNA Interactome (https://circinteractome.nia.nih.gov/) and TargetScan (http://www.targetscan.org/), respectively. The partial sequences of circ_0043265 or SALL1 3′-untranslated region (UTR), which contains the putative miR-1243-binding site, were amplified via PCR and constructed into the pmirGLO luciferase vector (Promega, Madison, WI, United States) to generate wild-type circ_0043265 reporter (circ_0043265-WT) or SALL1 reporter (SALL1-WT). The GeneArt™ Site-Directed Mutagenesis System (Thermo Fisher Scientific, Waltham, MA, United States) was used to produce miR-1243 target site-mutation circ_0043265 (circ_0043265-MUT) reporter or miR-1243 target site-mutation SALL1 3′-UTR (SALL1-MUT) reporter. All constructs were verified via DNA sequencing. Subsequently, the luciferase reporters and miR-1243 mimic or control mimic were co-transfected into SCC25 and Cal27 cells using Lipofectamine 2000. 48 h after transfection, cells were collected and the relative firefly luciferase activities were measured using a dual-luciferase reporter assay system (Promega, Madison, WI, United States) following the manufacturer's instructions.

### RNA Pull-Down Assay

RNA pull-down assay was performed as described previously [14]. Briefly, full length circ_0043265 and circ_0043265-MUT transcripts were transcribed from pmirGLO-WT-circ_0043265 and pmirGLO-MUT-circ_0043265, and linearized using the specific restriction enzyme *in vitro*. Then, Ribo™ RNA max-T7 RNA polymerase and Biotin RNA Labeling kit (RiboBio, Guangzhou, China) were used to produce biotin-labeled circ_0043265 and negative control biotinylated probe according to the manufacturer's instructions. Subsequently, 5 µg of biotin-labeled circ_0043265 or circ_0043265-MUT was incubated with 1 mg of cell lysates, which were lyzed using NP40 solution (Beyotime, Shanghai, China) at 4 °C for 4 h. Subsequently, the RNAs with biotin-labelled circ_0043265 or circ_0043265-MUT were mixed with 40 µL of Dynabeads^®^ MyOne™ Streptavidin C1 beads (Invitrogen/Thermo Fisher Scientific, Carlsbad, CA, United States) and incubated at 4 °C on a rotator overnight. Subsequently, the pulled-down RNA was identified via qRT-PCR analysis.

### Statistical Analysis

SPSS 22.0 statistical software (SPSS, Chicago, IL, United States) was used to perform statistical analysis. The data were presented as the mean ± standard deviation (SD). Student's t-test was used to compare the difference between the two groups, and ANOVA (Analysis of Variance) was used to compare the difference between multiple groups. Kaplan-Meier curve and log-rank tests were used to analyze the survival rates of different groups. Pearson's correlation analysis was used to analyze the correlations between circ_0043265, miR-1243 and SALL1 mRNA expression in TSCC tissues. *P* < 0.05 was considered to indicate a statistically significant difference.

## Results

### Circ_0043265 Expression Is Downmodulated in TSCC Tissues and Cell Lines

**FIGURE 1:**
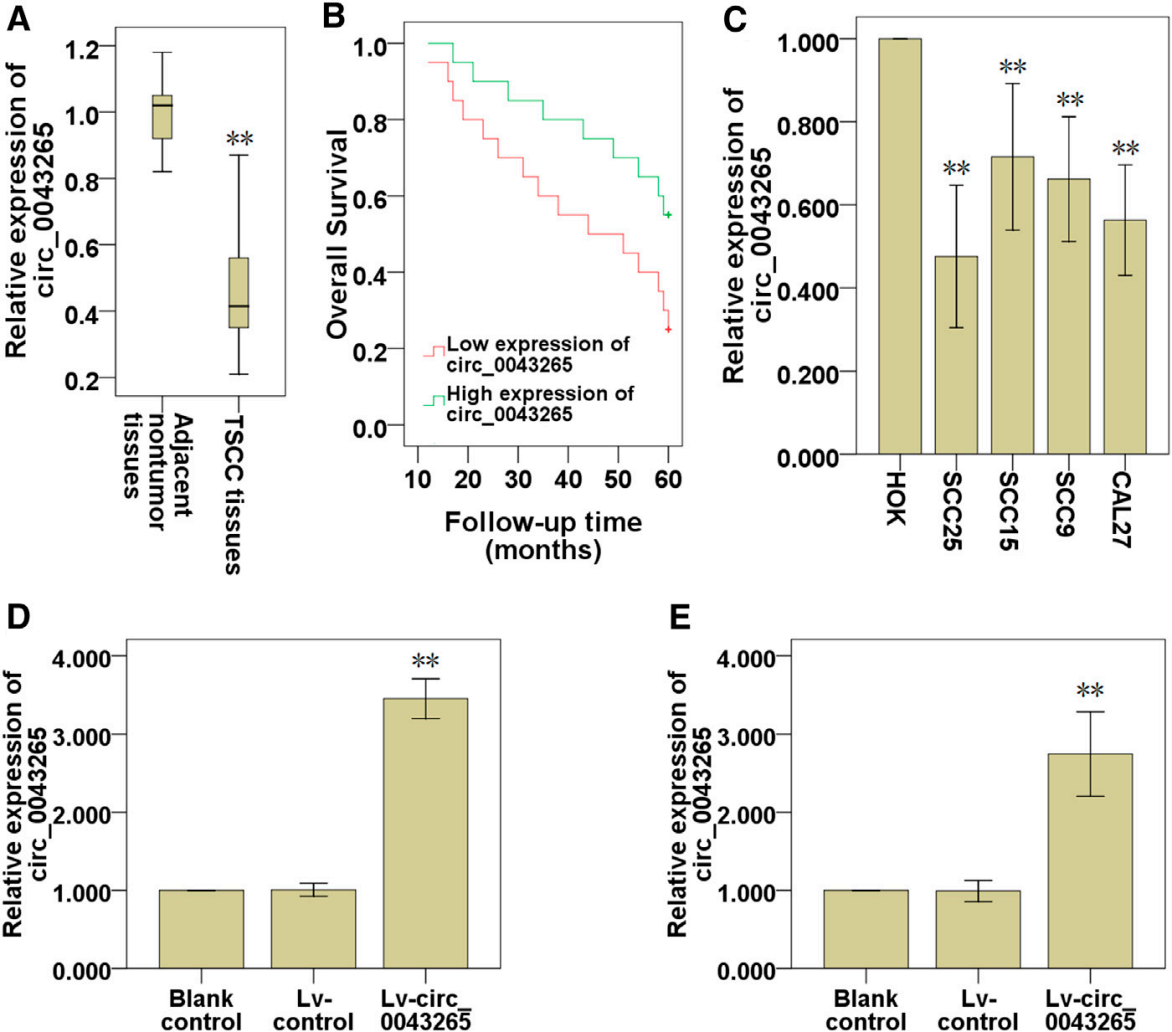
Circ_0043265 is downmodulated in TSCC tissues and cell lines. **(A)** Expression of circ_0043265 in TSCC tissues and their adjacent non-tumor tissues. **(B)** The survival curve of TSCC patients with low and high circ_0043265 expression based on median circ_0043265 level in TSCC tissues. **(C)** Relative expression levels of circ_0043265 in HOK and TSCC cell lines. The expression levels of circ_0043265 were upmodulated in SCC25 **(D)** and Cal27 **(E)** cells transfected with Lv-circ_0043265, as determined using qRT-PCR. **P< 0.01 vs. control (adjacent non-tumor tissues, HOK, Lv-control).

QRT-PCR analysis indicated that circ_0043265 was significantly downmodulated in TSCC tissues compared with their adjacent non-tumor tissues (Figure 1A). Subsequently, the prognostic value of circ_0043265 expression levels in TSCC tissues was investigated via analyzing TSCC patients’ clinical data. It was demonstrated that the overall survival (OS) of the TSCC patients exhibiting high circ_0043265 levels was significantly higher compared with those with low circ_0043265 levels (Figure 1B). When analyzing the correlation between circ_0043265 and clinicopathologic features of the patients, we just found that the expression of circ_0043265 had a downward trend in the advanced cases, but did not find any significant association between circ_0043265 expression and clinicopathological features in the 40 TSCC patients (Table 2). The lack of association may be a result of insufficient patient cohort. In addition, circ_0043265 levels were also significantly downmodulated in TSCC cell lines (SCC25, SCC15, SCC9 and Cal27) compared with HOK (Figure 1C). These above results suggested that circ_0043265 might be a potential tumor suppressor in TSCC. Since the expression of hsa_circ_0043265 in the two cell lines, SCC-25 and Cal-27, was relatively low, we chose the two cell lines for the gain-function experiment. Therefore, in a subsequent experiment, the circ_0043265 levels in SCC25 and Cal27 cells were upmodulated using Lv-circ_0043265 (Figures 1D,E), and then the effects of circ_0043265 on the cell proliferation, migration and invasion of SCC25 and Cal27 cells were further investigated.

**TABLE 2:** Correlation between circ_0043265 and clinicopathologic features of patients.

Clinicopathologic factors	All patients	Circ_0043265 expression		χ^2^	*p*-value
		High level	Low level		
Age					
≤55	18	8	10	0.404	0.525
>55	22	12	10		
Gender					
Male	23	12	11	0.102	0.749
Female	17	8	9		
TNM stage					
I + II	17	11	6	2.558	0.110
III + IV	23	9	14		
Size (cm)					
≤2	22	13	9	1.616	0.204
>2	18	7	11		

Note: The χ^2^ test was used for comparison between groups. Abbreviations: TNM, Tumor-Node-Metastasis.

### Effects of Circ_0043265 on the Cell Proliferation, Invasion and Migration of SCC25 and Cal27 Cells

**FIGURE 2:**
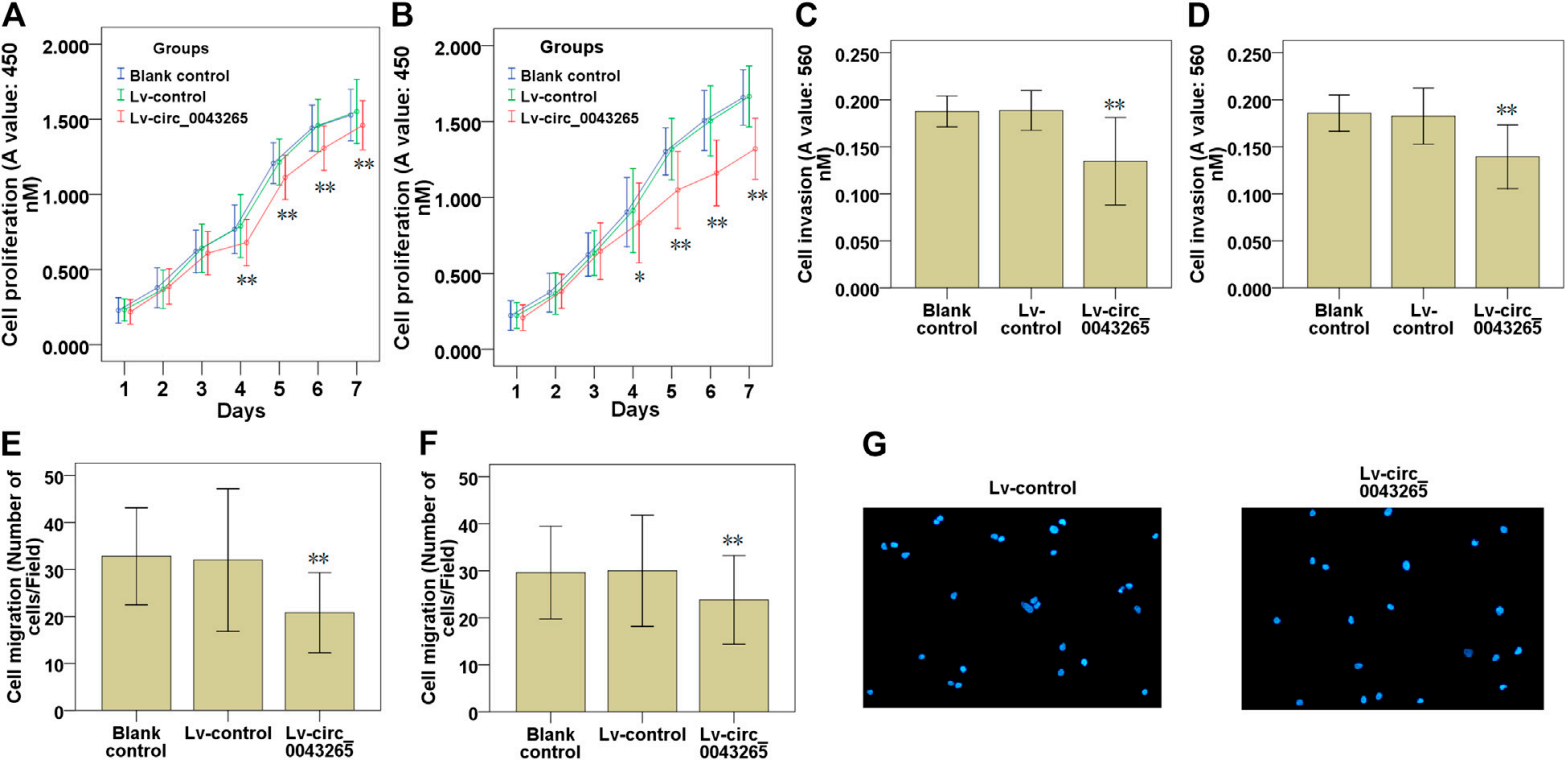
Effects of circ_0043265 overexpression on the cell proliferation, migration and invasion of TSCC cells. Effects of circ_0043265 overexpression on the cell proliferation of SCC25 **(A)** and Cal27 **(B)** cells, as determined using CCK-8. Effects of circ_0043265 overexpression on cell invasion of SCC25 **(C)** and Cal27 **(D)** cells, as determined via a Matrigel-coated Transwell assay. Effects of circ_0043265 overexpression on the cell migration of SCC25 **(E)** and Cal27 **(F)** cells, as determined via a Transwell assay. Representative pictures of the cell migration in SCC25 **(G)** cells were manifested. *P< 0.05, **P< 0.01 vs. Lv-control.

CCK-8 assays demonstrated that the cell proliferation of SCC25 and Cal27 cells transfected with Lv-circ_0043265 were significantly restrained compared with those of the cells transfected with Lv-control (Figures 2A,B). Subsequently, the effects of circ_0043265 on the cell invasion and migration of SCC25 and Cal27 cells were further explored. Cell invasion assays demonstrated that the invasive capability of SCC25 and Cal27 cells transfected with Lv-circ_0043265 were significantly decreased compared with cells transfected with Lv-control (Figures 2C,D). Cell migration assays also demonstrated that the numbers of migrating SCC25 and Cal27 cells transfected with Lv-circ_0043265 were significantly reduced compared with cells transfected with Lv-control (Figures 2E–G). Overall, these above results indicated that circ_0043265 overexpression restrained the cell proliferation, invasion and migration of TSCC cells.

### Circ_0043265 Is Inversely Correlated With MiR-1243 in TSCC

**FIGURE 3:**
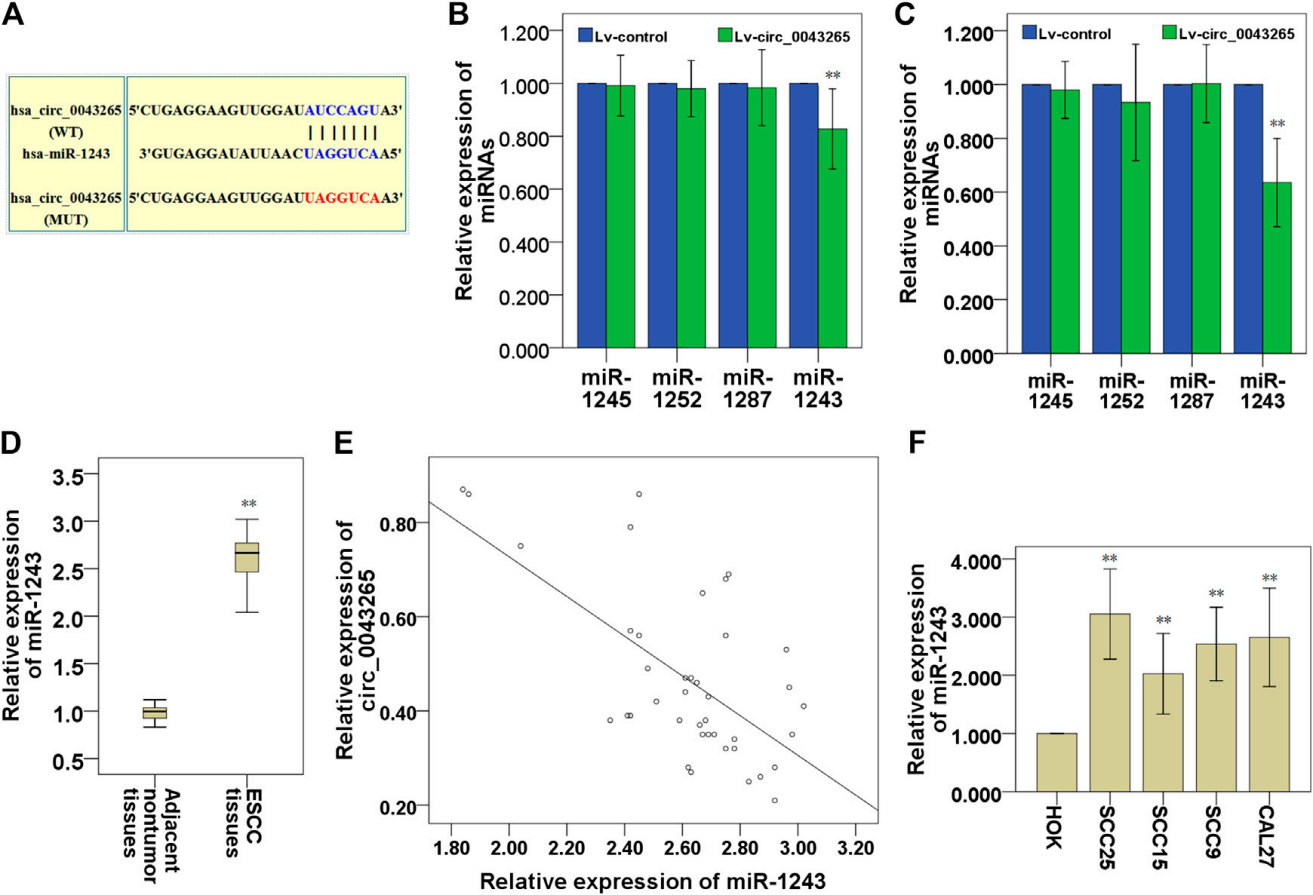
Circ_0043265 expression is inversely correlated with miR-1243 expression in TSCC. A predicted binding site of miR-1243 within circ_0043265 using Circular RNA Interactome. WT and MUT targeting regions of circ_0043265 for miR-1243 **(A)**. Effects of circ_0043265 overexpression on the expression of miRNAs that possessed putative binding sites for circ_0043265 in SCC25 **(B)** and Cal27 **(C)** cells. Expression levels of miR-1243 in TSCC and their adjacent non-tumor tissues **(D)**. Association between miR-1243 and circ_0043265 in TSCC tissues **(E)**. Expression levels of miR-1243 in TSCC cells and HOK **(F)**. **P< 0.01 vs. control (Lv-control, adjacent non-tumor tissues, HOK).

MiR-1245, miR-1252, miR-1287 and miR-1243 were identified as potential targeting miRNAs containing putative binding sites for circ_0043265 via a search of an online bioinformatics database (Circular RNA Interactome: https://circinteractome.nia.nih.gov/; Table 3). In this bioinformatics analysis, we found that miR-1243 may bind to the 3′-UTR region of SALL1 (Figure 3A). Subsequently, the expression levels of miR-1243 were demonstrated to be significantly downmodulated following overexpression of circ_0043265 in SCC25 and Cal27 cells, but the expression levels of miR-1245, miR-1252 and miR-1287 were not significantly altered following overexpression of circ_0043265 in SCC25 or Cal27 cells (Figures 3B,C). QRT-PCR analysis demonstrated that miR-1243 levels were significantly upmodulated in TSCC tissues compared with their adjacent non-tumor tissues (Figure 3D), and were inversely correlated with circ_0043265 levels (r = −0.663; Figure 3E). Furthermore, miR-1243 expression was also enhanced in TSCC cell lines (SCC25, SCC15, SCC9 and Cal27) compared with HOK (Figure 3F). Overall, these above results suggested that circ_0043265 may negatively modulate miR-1243 expression and function in TSCC.

**TABLE 3:** The predicted miRNAs targeting circ_0043265 sequence.

CircRNA Mirbase ID	CircRNA (top) - miRNA (bottom) pairing	Site type	CircRNA start	CircRNA end	3′ pairing	context + score percentile
hsa_circ_0043265 (5' ... 3′) hsa-miR-1245 (3' ... 5′)	UACAUUAAGAUGGCAGAUCACUA | | | | | | | | UACAUCCGGAAAUCUAGUGAA	8mer-1a	1,028	1,035	0.003	95
hsa_circ_0043265 (5' ... 3′) hsa-miR-1252 (3' ... 5′)	CCUUAUGGUAUGAAGUUCCUUCA | | | | | | | AUUUACUUAAGUUAAAGGAAGA	8mer-1a	57	64	0.003	98
hsa_circ_0043265 (5' ... 3′) hsa-miR-1287 (3' ... 5′)	UGCUACUAUUGCUACUCCAGCAG | | | | | | CUGAGCUUGGUGACUAGGUCGU	7mer-1a	1702	1708	0.004	92
hsa_circ_0043265 (5' ... 3′) hsa-miR-1243 (3' ... 5′)	CUGAGGAAGUUGGAUAUCCAGUA | | | | | | | GUGAGGAUAUUAACUAGGUCAA	8mer-1a	1,428	1,435	0.024	98

### Circ_0043265 Directly Downmodulates MiR-1243 Expression in TSCC Cells

**FIGURE 4:**
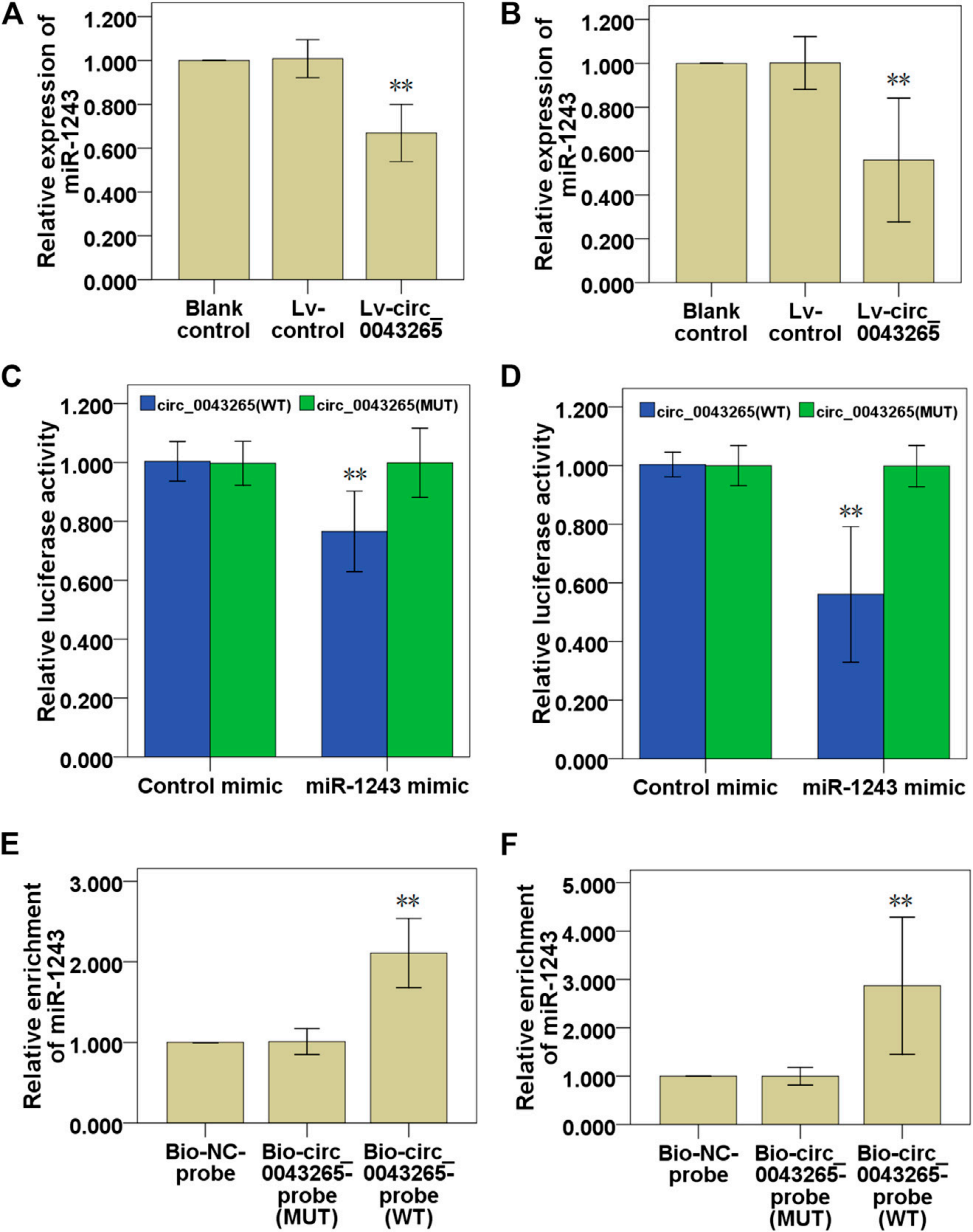
Circ_0043265 directly downmodulates miR-1243 expression in TSCC cells. Effects of circ_0043265 on the expression of miR-1243 in SCC25 **(A)** and Cal27 **(B)** cells. Relative luciferase activity in SCC25 **(C)** and Cal27 **(D)** cells co-transfected with circ_0043265-WT or circ_0043265-MUT luciferase plasmid and control mimic or miR-1243 mimic. Detection of circ_0043265 in RNA pulled down via Bio-circ_0043265, Bio-circ_0043265-MUT or Bio-NC probes. Detection of miR-1243 in RNA pulled down via Bio-circ_0043265, Bio-circ_0043265-MUT or Bio-NC probes in SCC25 **(E)** and Cal27 **(F)** cells. **P< 0.01 vs. control (Lv-control, control mimic, Bio-NC-probe).

First, it was demonstrated that miR-1243 expression levels were significantly downmodulated in SCC25 and Cal27 cells transfected with Lv-circ_0043265 compared with the cells transfected with Lv-control (Figures 4A,B). Dual-luciferase reporter assays indicated that the relative luciferase activities in SCC25 and Cal27 cells co-transfected with circ_0043265-WT and miR-1243 mimic were significantly decreased compared with cells transfected with control mimic (Figures 4C,D). By contrast, co-transfecting circ_0043265-MUT and miR-1243 mimics into SCC25 and Cal27 cells did not significantly affect the relative luciferase activity (Figures 4C,D). Furthermore, biotin-labeled RNA pulldown assays demonstrated that a bio-circ_0043265 probe could directly pull down miR-1243, but the associated bio-circ_0043265-MUT-probe failed to pull down miR-1243 in SCC25 or Cal27 cells (Figures 4E,F). Overall, these above results indicated that circ_0043265 could directly downmodulate miR-1243 at special recognition sites in TSCC cells.

### Circ_0043265 Acts as a ceRNA to Restrain the Cell Proliferation, Invasion and Migration of SCC25 and Cal27 Cells via Directly Sponging MiR-1243

**FIGURE 5:**
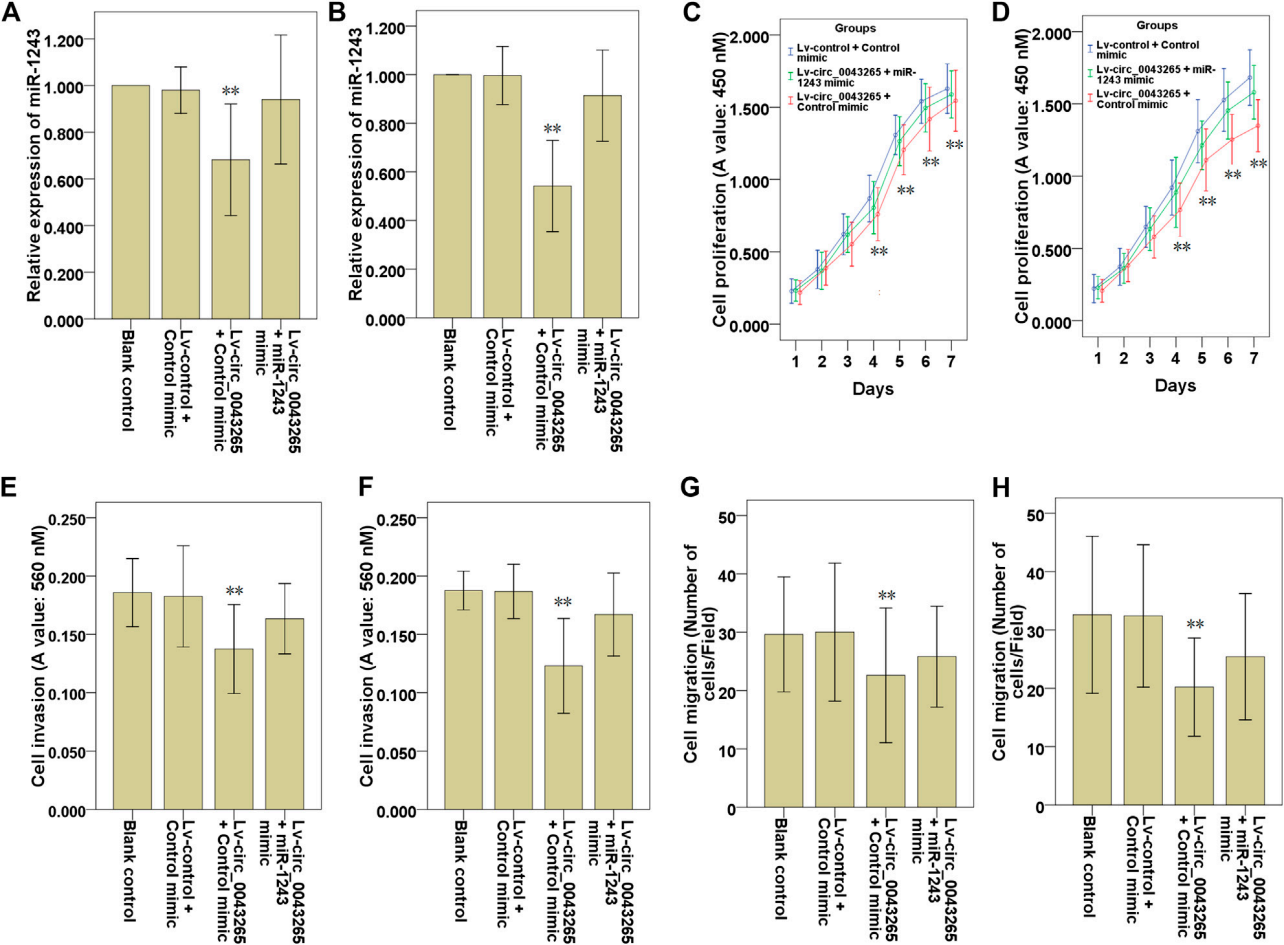
Upmodulation of miR-1243 reverses the effects of circ_0043265 on the cell proliferation, invasion and migration of TSCC cells. Expression of miR-1243 in SCC25 **(A)** and Cal27 **(B)** cells transfected with Lv-control + control mimic, Lv-circ_0043265 + control mimic or Lv-circ_0043265 + miR-1243 mimics, as determined via qRT-PCR. Proliferation of SCC25 **(C)** and Cal27 **(D)** cells transfected with Lv-control + control mimic, Lv-circ_0043265 + control mimic or Lv-circ_0043265 + miR-1243 mimics, as determined via a CCK-8 assay. The cell invasion ability of SCC25 **(E)** and Cal27 **(F)** cells transfected with Lv-control + control mimic, Lv-circ_0043265 + control mimic or Lv-circ_0043265 + miR-1243 mimics, as determined via a Matrigel-coated Transwell assay. The cell migration ability of SCC25 **(G)** and Cal27 **(H)** cells transfected with Lv-control + control mimic, Lv-circ_0043265 + control mimic or Lv-circ_0043265 + miR-1243 mimics, determined via a Transwell assay. **P< 0.01 vs. Lv-control + control mimic.

First, it was confirmed that the decreased miR-1243 expression level resulted from circ_0043265 overexpression, and that recovery of the expression of miR-1243 was identified following co-transfection with miR-1243 mimic in SCC25 and Cal27 cells (Figures 5A,B). Subsequently, CCK-8 assays were employed to investigate the effects of Lv-circ_0043265 on the cell proliferation of SCC25 and Cal27 cells, and these inhibitory effects were rescued via co-transfection of miR-1243 mimic (Figures 5C,D). Similarly, Transwell assays demonstrated that upmodulation of miR-1243 could reverse the inhibitory effects of Lv-circ_0043265 on cell invasion and migration of SCC25 and Cal27 cells (Figures 5E–H).

### Circ_0043265 Facilitates SALL1 Expression via Modulating MiR-1243

**FIGURE 6:**
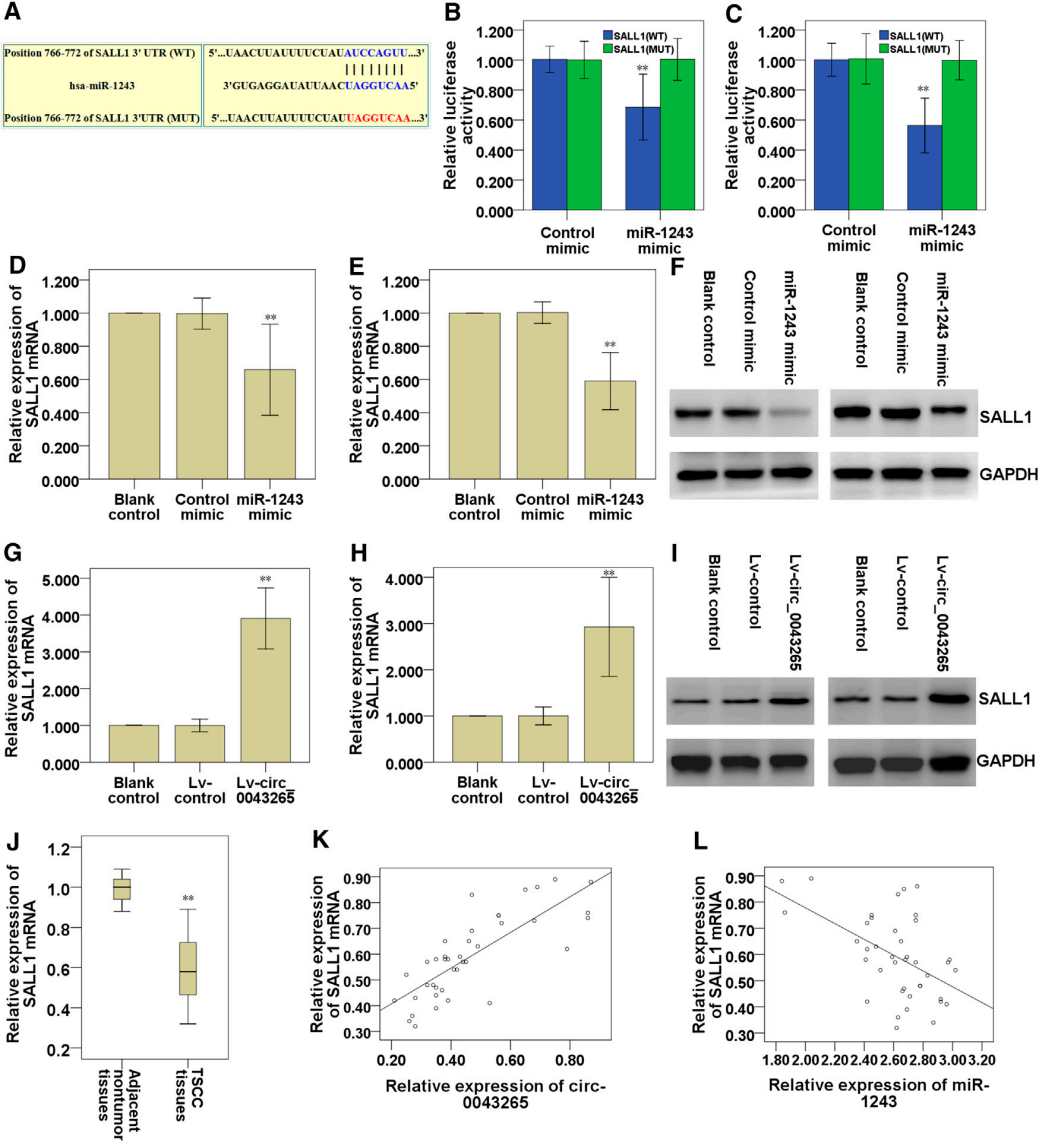
Circ_0043265 facilitates SALL1 expression via sponging miR-1243. A predicted binding site of miR-1243 within the SALL1 3′-UTR region using Targetscan **(A)**. Luciferase reporter assays were used to evaluate the interaction between SALL1 3′-UTR and miR-1243 in SCC25 **(B)** and Cal27 **(C)** cells. Relative mRNA levels of SALL1 in miR-1243-overexpressed SCC25 **(D)** and Cal27 **(E)** cells were measured using qRT-PCR. Relative protein levels of SALL1 in miR-1243-overexpressed SCC25 and Cal27 cells were measured using western blots **(F)**. Relative mRNA levels of SALL1 in circ_0043265-overexpressed SCC25 **(G)** and Cal27 **(H)** cells were measured using qRT-PCR. Relative protein levels of SALL1 in circ_0043265-overexpressed SCC25 and Cal27 cells were measured using western blots **(I)**. Expression levels of SALL1 mRNA in TSCC and the adjacent non-tumor tissues **(J)**. Spearman correlation analysis between SALL1 and circ_0043265 expressions in TSCC tissues **(K)**. Spearman correlation analysis between SALL1 and miR-1243 expressions in TSCC tissues **(L)**. **P< 0.01 vs. control (Control mimic, Lv-control, adjacent non-tumor tissues).

The predicted targets for miR-1243 were identified using miRDB (http://mirdb.org/), and the bioinformatics analysis predicted that SALL1 may be a direct target of miR-1243 (Table 4). Thus, we further analyzed the binding partner of miR-1243 using Targetscan (http://www.targetscan.org/vert_72/), and found that miR-1243 may bind to the 3′-UTR region of SALL1 (Figure 6A). Luciferase assays confirmed that miR-1243 could bind to SALL1, and this binding restrained SALL1 luciferase activity in SCC25 and Cal27 cells (Figures 6B,C). Furthermore, overexpression of miR-1243 in SCC25 and Cal27 cells significantly restrained SALL1 mRNA and protein expression (Figures 6D–F), further confirming that miR-1243 restrained SALL1 expression. We also found that the overexpression of circ_0043265 facilitated both mRNA and protein levels of SALL1 (Figures 6G–I), suggesting that circ_0043265 positively modulated SALL1 expression. Indeed, SALL1 was lowly expressed in TSCC tissues (Figure 6J); SALL1 and circ_0043265 were positively correlated (r = 0.817; Figure 6K), and meanwhile SALL1 and miR-1243 expressions were inversely associated in TSCC sample tissues (r = −0.515; Figure 6L). Overall, these above results indicated that circ_0043265 elevated SALL1 expression via modulating miR-1243.

**TABLE 4:** The predicted targets for hsa-miR-1243 in miRDB.

Target rank	Target score	MiRNA name	Gene symbol	Gene description
1	97	Hsa-miR-1243	*CLUL1*	Clusterin like 1
2	97	Hsa-miR-1243	*ASB7*	Ankyrin repeat and SOCS box containing 7
3	97	Hsa-miR-1243	*OSBPL11*	Oxysterol binding protein like 11
4	96	Hsa-miR-1243	*MAP3K5*	Mitogen-activated protein kinase 5
5	95	Hsa-miR-1243	*RAP1B*	RAP1B, member of RAS oncogene family
6	95	Hsa-miR-1243	*TBL1XR1*	Transducin beta like 1 X-linked receptor 1
7	94	Hsa-miR-1243	*STC2*	Stanniocalcin 2
8	94	Hsa-miR-1243	*ERC2*	ELKS/RAB6-interacting/CAST family member 2
9	94	Hsa-miR-1243	*HNRNPH2*	Heterogeneous nuclear ribonucleoprotein H2
10	92	Hsa-miR-1243	*TNS3*	Tensin 3
11	91	Hsa-miR-1243	*HIST2H2BF*	Histone cluster 2 H2B family member f
12	91	Hsa-miR-1243	*SALL1*	Spalt like transcription factor 1
13	91	Hsa-miR-1243	*GNPDA1*	Glucosamine-6-phosphate deaminase 1
14	91	Hsa-miR-1243	*MYOZ1*	Myozenin 1
15	90	Hsa-miR-1243	*ZNF382*	Zinc finger protein 382
16	90	Hsa-miR-1243	*TAGAP*	T Cell activation RhoGTPase activating protein
17	90	Hsa-miR-1243	*TRIM4*	Tripartite motif containing 4

### SALL1 Knockdown Ameliorates the Inhibitory Effects of Circ_0043265 on Cell Proliferation, Invasion and Migration of TSCC Cells

**FIGURE 7:**
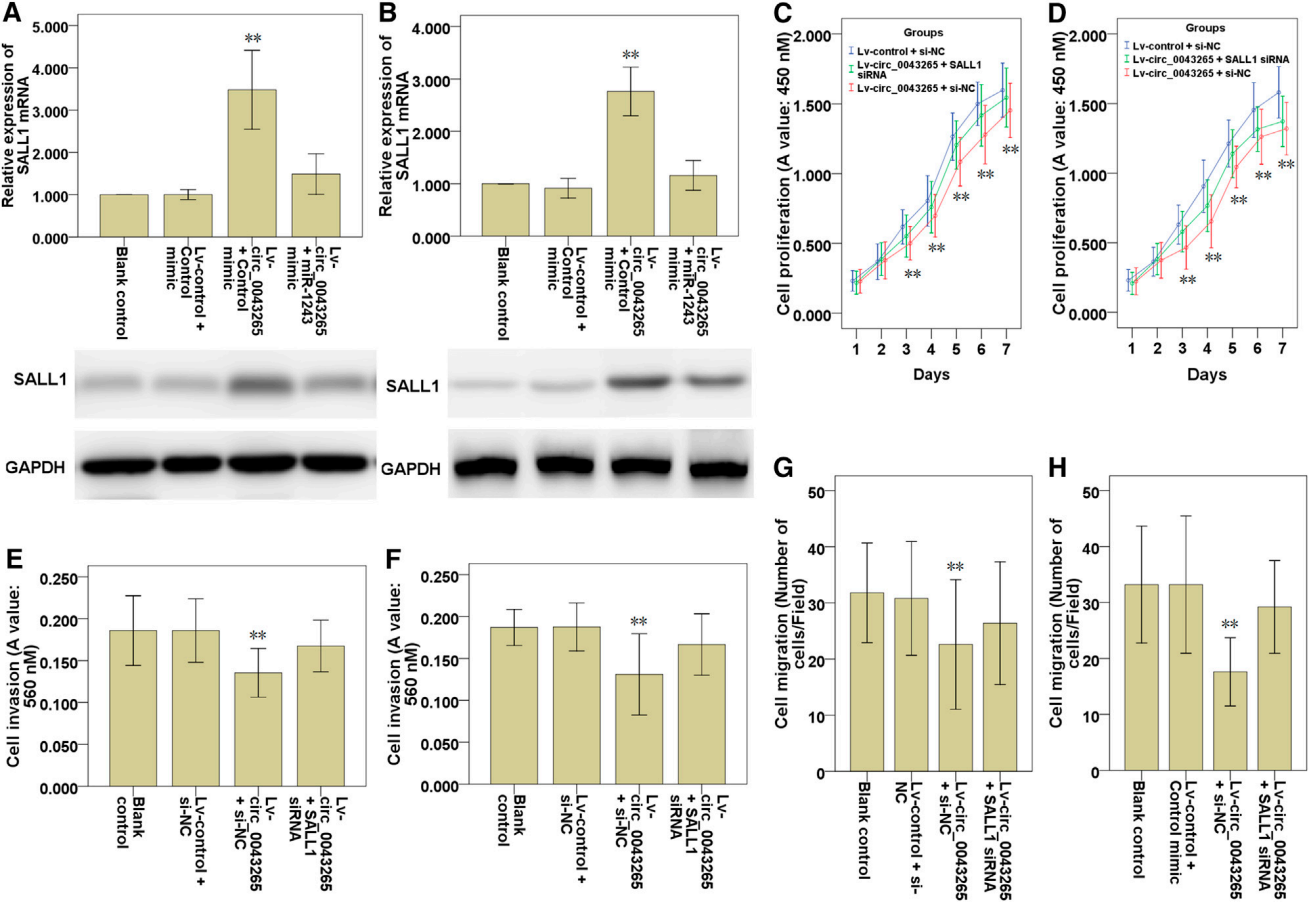
Knockdown SALL1 reverses the inhibitory effects of circ_0043265 on the cell proliferation, migration and invasion of TSCC cells. SALL1 mRNA and protein expression in SCC25 **(A)** and Cal27 **(B)** cells transfected with Lv-control + control mimic, Lv-circ_0043265 + control mimic or Lv-circ_0043265 + miR-1243 mimics. Cell proliferation of SCC25 **(C)** and Cal27 **(D)** cells transfected with Lv-control + siRNA NC (si-NC), Lv-circ_0043265 + si-NC or Lv-circ_0043265 + SALL1 siRNA, as determined via a CCK-8 assay. The cell invasion ability of SCC25 **(E)** and Cal27 **(F)** cells transfected with Lv-control + si-NC, Lv-circ_0043265 + si-NC, or Lv-circ_0043265 + SALL1 siRNA, as determined via a Matrigel-coated Transwell assay. The cell migration ability of SCC25 **(G)** and Cal27 **(H)** cells transfected with Lv-control + si-NC, Lv-circ_0043265 + si-NC, or Lv-circ_0043265 + SALL1 siRNA, as determined via a Transwell assay. **P< 0.01 vs. Lv-control + control mimic or si-NC.

The effects of circ_0043265 on the mRNA and protein expression levels of SALL1 were examined, and both were demonstrated to be upmodulated as a consequence of circ_0043265 overexpression, although the mRNA and protein expression levels were subsequently decreased following co-transfection of miR-1243 mimic in SCC25 and Cal27 cells (Figures 7A,B), suggesting that circ_0043265 overexpression upmodulated SALL1 expression via miR-1243. In functional terms, the cell proliferation, invasion and migration of SCC25 and Cal27 cells infected with Lv-circ_0043265 were restored following the knockdown of SALL1 (Figures 7C–H); the effects on the cell proliferation are presented in Figures 7C,D, the effects on the cell invasion are presented in Figures 7E,F, whereas those on the cell migration are presented in Figures 7G,H. Overall, these above results indicated that circ_0043265 restrained the cell proliferation, invasion and migration of TSCC cells via the miR-1243/SALL1 axis.

## Discussion

**FIGURE 8:**
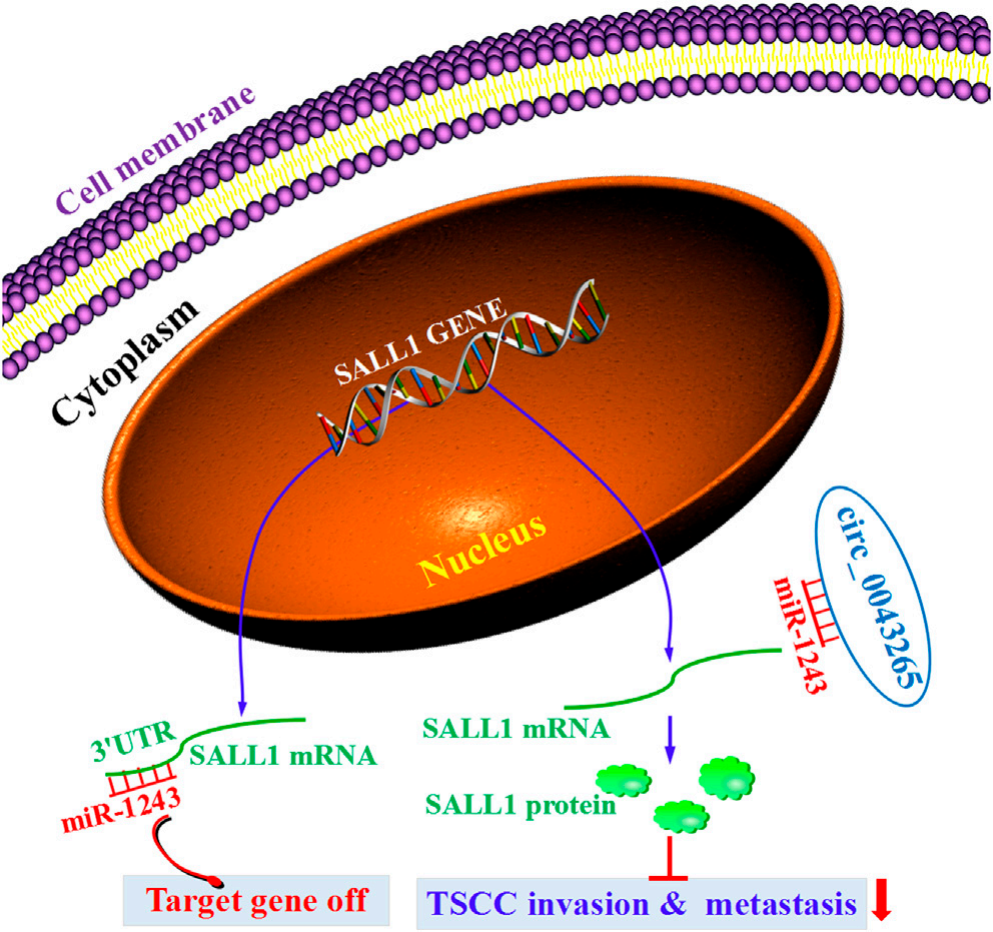
The diagrammatic sketch revealing the tumorigenesis-inhibiting role of circ_0043265 via regulation of miR-1243/SALL1 axis in TSCC.

CircRNAs have been identified as crucial regulators and biomarkers in TSCC initiation and progression [7–11]. In the current study, it was demonstrated that circ_0043265 was downmodulated in TSCC tissues and cell lines compared with their normal counterparts. Circ_0043265 expression was demonstrated to be positively associated with the overall survival rate of TSCC patients. However, we did not find any significant association between circ_0043265 expression and clinicopathological features in the TSCC patients. The lack of association may be a result of insufficient patient cohort, which should be further confirmed in larger TSCC patient cohort. Additionally, circ_0043265 overexpression restrained the cell proliferation, migration and invasion via targeting miR-1243/SALL1 axis in TSCC cells. Here, the first evidence was provided that circ_0043265 is able to sponge miR-1243 and upmodulate the expression of SALL1, a direct target of miR-1243 in TSCC. Collectively, these results indicated that circ_0043265 may act as a ceRNA to restrain TSCC invasion and metastsis via targeting the miR-1243/SALL1 axis (Figure 8).

A recently published study reported that hsa_circ_0043265 is lowly expressed in NSCLC tissues compared to adjacent normal tissues screened via a circRNA microarray [15]. Another previous study reported that circ_0043265 could sponge miR-25–3p to improve FOXP2 expression, thereby restraining NSCLC progression [12]. Consistent with the previous studies, our current study showed that circ_0043265 is lowly expressed in TSCC tissues and cell lines, and circ_0043265 overexpression restrained the cell proliferation, cell migration and invasion in TSCC cells, suggesting that circ_0043265 could act as a tumor suppressor in TSCC. Nevertheless, the underlying mechanism of circ_0043265's suppressive role in TSCC initiation and progression is yet to be fully determined.

CeRNA regulatory networks are commonly associated with the initiation and progression of various tumors [16,17] including TSCC [18,19]. Further, an increasing body of evidence has demonstrated that circRNAs are able to act as ceRNAs to interfere with miRNAs and their downstream target genes [20,21]. For instance, circRNA circNTRK2 facilitated the initiation and progression of esophageal squamous cell carcinoma (ESCC) via the miR-140–3p/NRIP1 axis [22]. In addition, circRNA circ-ZEB1, acting as a ceRNA to modulate the expression of miR-448, facilitated the cell proliferation and invasion of triple negative breast cancer cells [23]. In the current study, we predicted and confirmed that miR-1243 was a downstream target miRNA for circ_0043265. MiR-1243 was identified as a potential targeting miRNA that possessed putative binding sites for circ_0043265. Subsequently, it was revealed that circ_0043265 could directly sponge and bind to miR-1243 at the special recognition sites, acting as a ceRNA to modulate the expression and function of miR-1243. Thus, the function and mechanism of miR-1243 in circ_0043265's suppressive role in TSCC was then investigated.

MiR-1243 has been demonstrated to be involved in the initiation and progression in multiple types of cancer, such as pancreatic cancer and osteosarcoma [24,25]. It is associated with the roles of oncogenic or tumor-suppressive genes, depending on the tumor type involved. For instance, miR-1243 restrained cell migration and invasion and targeted EMT-related genes in pancreatic cancer cells [25]. By contrast, miR-1243 expression was confirmed to be upmodulated in osteosarcoma cells [24]. In the current study, it was demonstrated that the suppressive effects of circ_0043265 on cell proliferation, migration and invasion of TSCC cells were rescued via miR-1243 overexpression. Therefore, combining the results observed in previous studies with our current findings supports the hypothesis that circ_0043265 acts as a ceRNA to restrain the malignant phenotypes of TSCC cells via supressing the miR-1243.

In the next part of our study, we further explored how miR-1243 interacted with the downstream potential target genes. Since the function of miRNAs are realized via modulating their target genes’ mRNA expression [26], we here conducted the bioinformatics analysis and verification experiment, and revealed that spalt like transcription factor 1 (SALL1) was one of the potential downstream target genes of miR-1243. The SALL1 is a member of Krüppel-associated box-containing zinc finger proteins (KRAB-ZFPs), and has been revealed to modulate the initiation and progression of human tumors [27,28]. SALL1 acted as a tumor suppressor gene in human glioma [27] and breast cancer (Ma et al., 2018). Further, miR-4286 may exert its tumor-promoting actions, in part, by downmodulating SALL1 in prostate cancer (PCa) [28]. Based on the above studies, we hypothesized that SALL1 may be partially required for circ_0043265 to exert its anti-oncogenic effect in TSCC. To address this point, the current rescue experiment revealed that knockdown of SALL1 partly blocked circ_0043265 restoration-induced suppression of cell proliferation, migration and invasion in TSCC cells. Hence, these results suggested that circ_0043265 may restrain the malignant phenotypes of TSCC cells via modulating SALL1.

Taken together, it was demonstrated that circ_0043265 is a potential tumor suppressor in TSCC. Circ_0043265 could restrain cell proliferation, cell migration and invasion via modulating miR-1243 and SALL1 in TSCC cells. In investigating the underlying mechanism, the current study demonstrated that circ_0043265 acted as a sponge of miR-1243 to facilitate SALL1 expression. The current study has provided novel insight into the regulatory mechanisms of the circ_0043265/miR-1243/SALL1 axis in TSCC, and may provide a novel biomarker or target for the diagnosis and treatment of TSCC in the future.

## Data Availability

The datasets presented in this study can be found in online repositories. The names of the repositories and accession numbers can be found in the article.
